# Supplementary material for: The Influence of Caerulomycin A on the Intestinal Microbiota in SD Rats
Source: Mar Drugs. 2020 May 22;18(5):277. doi: 10.3390/md18050277 (PMC7281470; doi:10.3390/md18050277)
Supplement: Supplementary file 1 [file marinedrugs-18-00277-s001.pdf]

Table 1. the tags of the samples.

Sample_ID	clean_tags	valid_tags	valid_percent	valid minLength	valid meanLength	valid maxLength	subsample_depth	OTU_counts	Total_OTUs
HDZWM954.0.1	37571	34157	90.91%	243	432.53	443	13877	958	3980
HDZWM954.0.2	37432	33822	90.36%	243	431.36	446	13877	1291	3980
HDZWM954.0.3	39577	35518	89.74%	243	430.13	474	13877	1152	3980
HDZWM954.0.4	34721	31381	90.38%	243	429.04	474	13877	1031	3980
HDZWM954.0.5	36248	33262	91.76%	242	432.87	474	13877	947	3980
HDZWM954.0.6	36365	32477	89.31%	241	430.2	474	13877	1160	3980
HDZWM954.0.7	19846	17347	87.41%	243	434	443	13877	887	3980
HDZWM954.0.8	37735	33918	89.88%	241	429.65	442	13877	1166	3980
HDZWM954.0.9	36788	33068	89.89%	243	430.02	468	13877	1128	3980
HDZWM954.0.10	48301	43994	91.08%	239	430.76	474	13877	1131	3980
HDZWM954.2W.1	50143	46102	91.94%	239	430.81	442	13877	1129	3980
HDZWM954.2W.2	43394	39171	90.27%	240	432.27	474	13877	1102	3980
HDZWM954.2W.3	43506	40110	92.19%	239	431.73	474	13877	1082	3980
HDZWM954.2W.4	37642	33806	89.81%	241	427.37	445	13877	1207	3980
HDZWM954.2W.5	41111	36779	89.46%	243	429.02	474	13877	1199	3980
HDZWM954.2W.6	43350	39380	90.84%	241	429.81	474	13877	1222	3980
HDZWM954.2W.7	40234	36436	90.56%	243	431.49	474	13877	1292	3980
HDZWM954.2W.8	39018	34883	89.40%	243	429.03	445	13877	1117	3980
HDZWM954.2W.9	41981	38573	91.88%	241	432.36	443	13877	1155	3980
HDZWM954.2W.10	37264	33493	89.88%	243	431.12	474	13877	1197	3980
HDZWM954.3W.1	36670	33017	90.04%	240	430.84	474	13877	1032	3980
HDZWM954.3W.2	36269	32381	89.28%	243	429.33	474	13877	1013	3980
HDZWM954.3W.3	41241	37527	90.99%	243	431.94	474	13877	916	3980
HDZWM954.3W.4	25777	23581	91.48%	243	431.6	474	13877	951	3980
HDZWM954.3W.5	40962	36986	90.29%	243	431.07	474	13877	1115	3980
HDZWM954.3W.6	37100	33677	90.77%	243	430.83	474	13877	1159	3980
HDZWM954.3W.7	46488	41862	90.05%	243	429.81	474	13877	1111	3980
HDZWM954.3W.8	41035	36583	89.15%	241	430.28	445	13877	871	3980
HDZWM954.3W.9	39758	36754	92.44%	239	430.66	474	13877	908	3980
HDZWM954.3W.10	41466	37935	91.48%	240	430.33	474	13877	877	3980
HDZWM954.4W.1	51139	46046	90.04%	241	429.96	474	13877	1082	3980
HDZWM954.4W.2	36943	33279	90.08%	241	429.49	474	13877	1154	3980
HDZWM954.4W.3	48727	43573	89.42%	242	431.27	442	13877	1203	3980
HDZWM954.4W.4	49569	44866	90.51%	243	430.77	450	13877	1196	3980
HDZWM954.4W.5	49483	43828	88.57%	241	429.88	474	13877	1109	3980
HDZWM954.4W.6	40780	35557	87.19%	243	431.67	442	13877	1155	3980
HDZWM954.4W.7	47256	42476	89.88%	241	430.64	457	13877	1186	3980
HDZWM954.4W.8	32785	29106	88.78%	243	430.76	445	13877	1188	3980
HDZWM954.4W.9	51218	46022	89.86%	243	430.06	474	13877	1092	3980
HDZWM954.4W.10	40776	36634	89.84%	242	429.98	474	13877	1167	3980
